# Single-Cell mRNA Sequencing in Cancer Research: Integrating the Genomic Fingerprint

**DOI:** 10.3389/fgene.2017.00073

**Published:** 2017-05-31

**Authors:** Sören Müller, Aaron Diaz

**Affiliations:** ^1^Department of Neurological Surgery, University of California, San Francisco, San FranciscoCA, United States; ^2^Eli and Edythe Broad Center of Regeneration Medicine and Stem Cell Research, University of California, San Francisco, San FranciscoCA, United States

**Keywords:** cancer genomics, single-cell sequencing, tumor microenvironment, cancer phylogenetics, cancer stem cells

## Abstract

Critical cancer mutations are often regional and mosaic, confounding the efficacy of targeted therapeutics. Single cell mRNA sequencing (scRNA-seq) has enabled unprecedented studies of intra-tumor heterogeneity and its role in cancer progression, metastasis, and treatment resistance. When coupled with DNA sequencing, scRNA-seq allows one to infer the *in vivo* impact of genomic alterations on gene expression. This combination can be used to reliably distinguish neoplastic from non-neoplastic cells, to correlate paracrine-signaling pathways between neoplastic cells and stroma, and to map expression signatures to inferred clones and phylogenies. Here we review recent advances in scRNA-seq, with a special focus on cancer. We discuss the challenges and prospects of combining scRNA-seq with DNA sequencing to assess intra-tumor heterogeneity.

## Background

Next-generation sequencing (NGS) based studies have identified critical genetic alterations in a variety of malignancies (Brennan et al., 2013; Hoadley et al., 2014; Bai et al., 2015; Furnari et al., 2015; Ceccarelli et al., 2016; Cancer Genome Atlas Research Network, et al., 2016; Wang J. et al., 2016). However, relatively few targeted therapeutics are curative. Intra-tumor heterogeneity has emerged as an essential parameter confounding the delivery of a complete treatment (Saunders et al., 2012). Assessing tumor heterogeneity from bulk RNA or DNA extractions is limited to either inter-tumor comparisons (Cancer Genome Atlas Research Network, 2008; Müller et al., 2015), or comparisons across a small number of stereotactic biopsies (Gerlinger et al., 2012). Obtaining multi-region biopsies during complex cancer surgeries represents a major challenge. Moreover, inferring the contribution of specific tumor sub-clones and/or stromal cell-types from these data is a computationally difficult task, with a degree of uncertainty (El-Kebir et al., 2015; Turajlic et al., 2015).

The past decade has seen rapid advances in protocols for the faithful reverse-transcription (RT) and amplification of RNA from individual cells (Tang et al., 2009; Zong et al., 2012; Chapman et al., 2015). Microfluidic and other methods for single-cell isolation and library preparation have brought high-throughput single-cell RNA-sequencing (scRNA-seq) into the mainstream (Patel et al., 2014; Ting et al., 2014; Kim et al., 2015; Min et al., 2015; Hou et al., 2016; Li et al., 2016; Tirosh et al., 2016b; Gerber et al., 2017; Winterhoff et al., 2017). The limitations and uses of these novel data are still being defined. However, most state-of-the-art algorithms for analyzing sequencing data were not designed with single-cell studies in mind (Stegle et al., 2015; Bacher and Kendziorski, 2016).

Computational biologists are racing to keep up (Tanay and Regev, 2017). Novel analysis tools for single-cell cancer studies are being rapidly developed (Garmire et al., 2016). Already, scRNA-seq has led to groundbreaking insights into clonal tumor evolution (Navin et al., 2011), metastatic dissemination (Lawson et al., 2015), the development of chemo-resistance (Kim et al., 2016), and interactions between tumor and stromal cells (Choi et al., 2015). In this review, we summarize current advances in the acquisition and analysis of scRNA-seq data from samples of tumor tissue. We focus on the integration of orthogonal assays and future directions for scRNA-seq in cancer research.

## Single-Cell RNA Sequencing in Complex Tumor-Tissue

With only approximately 1pg of RNA in a single cell, and a median transcript abundance of fewer than 100 copies per gene, unbiased library-generation from such a small amount of starting material is challenging (Macaulay and Voet, 2014). The technical limitations of library generation for single-cell RNA-seq include non-uniform transcript coverage (3′ bias), and non-linear library amplification. Strategies are being actively developed to minimize these effects (Kolodziejczyk et al., 2015).

Two recent publications compared protocols for single-cell library-generation (Svensson et al., 2016; Ziegenhain et al., 2017). Both papers compared sensitivity and accuracy across protocols, using synthetic-RNA spike-in controls as a gold-standard. The advantage of using a gold-standard is the ability to assess not just sensitivity (which could be inferred without spike-in controls), but also accuracy. If cDNA copy-number accurately reflects mRNA abundance in single cells, then these data can be used quantitatively to compare expression within and between individual cells. The caveat of these studies is that synthetic-RNA spike-in controls are subject to library-preparation effects, treatment effects and other technical biases not observed when cloning cDNA from tissue-derived RNA (Risso et al., 2014).

Nonetheless, these two studies found that Smart-Seq2 was the most sensitive method. For example, almost twice as many genes per cell were detected via Smart-Seq2 when compared to Drop-seq, given similar sequencing depths. These two approaches represent two ends of the spectrum, in terms of trade-offs between transcriptome coverage and number of cells profiled. Methods that use standard oligo-dT primers (e.g., Smart-seq2), as compared to mRNA-capture beads (e.g., Drop-seq), have greater transcriptome coverage in terms of the number of distinct genes-sequenced, and greater coverage of the 5′ end of individual transcripts. The latter is especially useful when studying expressed mutations in cancer samples. Protocols like Smart-seq2 are typically applied in multi-well plate or microfluidic-chip based platforms that have a throughput of hundreds of cells. Droplet-based methods capture thousands of cells at a time.

Not surprisingly, batch effects have been observed when comparing batches of cells captured in separate assays (Tung et al., 2017). Specifically, the proportion of measured genes typically accounts for the major proportion of observed variability between batches (Hicks et al., under review). Best practices of experimental design, such as randomized blocking, are advised whenever possible. Statistical methods can also be used to adjust for batch effects *a posteriori* (Finak et al., 2015).

The effect of tissue dissociation on the efficiency of single-cell cDNA-library generation remains poorly understood. Some cell-isolation protocols for scRNA-seq may be biased toward certain cell types. For example, microfluidic platforms for automated library construction use chips which are graded to isolate cells of a given size (Müller et al., 2016). Biases present in droplet-based scRNA-seq platforms, for or against certain cell types, have not yet been fully investigated. Tumor disassociation protocols often involve cell selection by straining and/or density gradients (Venteicher et al., 2017). Fluorescence-activated cell sorting approaches to cell isolation followed by library preparation via Smart-seq2 provide perhaps the most flexible approach to apply scRNA-seq a specific, tumor-infiltrating cell-type of interest.

With the advent of droplet-based methods, there has been a trend to sequence more cells at lower coverage. This leads to a lower library-complexity per cell, and gives rise to the question: how many cells are required to obtain representative results from scRNA-seq data? As little as 50 cells have been shown to be sufficient to achieve a per-gene coefficient-of-variation that is comparable to a standard bulk RNA-seq experiment when sequencing a cell line (Shapiro et al., 2013). In another recent scRNA-seq study, only five cells from a patient-derived xenograft were required to represent 70% of the genes found in a bulk extraction (Kim et al., 2015), and robust transcriptome-wide correlations between single-cell and bulk experiments were observed when the sample sizes were increased to 35–50 cells. However, in both examples, cells were derived from relatively homogeneous populations.

Sample-size estimation in complex tissue, such as biopsies of patient tumors with a high degree of stromal infiltrate, remains an open problem. Given the wide range in cellular heterogeneity across cancer types, a one-size-fits-all recommendation as to sample size is likely impossible. However, techniques from capture statistics can be used to estimate sample sizes *ad hoc*, from pilot studies (Daley and Smith, 2013). Standards for sequencing depth per cell and methods to assess single-cell library complexity are beginning to emerge (Daley and Smith, 2014; Wu et al., 2014; Grün and Van Oudenaarden, 2015; Bacher and Kendziorski, 2016; Diaz et al., 2016). The majority of genes expressed in a cell are detected at a read-depth of 250,000–500,000 reads (Wu et al., 2014; Bacher and Kendziorski, 2016). If the goal is to survey cell diversity in an unbiased fashion, classify cell types by expression profile, and infer the proportions of each cell type, then even 50,000 reads per cell have been shown to be sufficient (Pollen et al., 2014). On the other hand, greater depth of coverage per cell is required to rigorously distinguish neoplastic from stromal cells, or to triage cells by the presence or absence of expressed mutations. We now discuss how low sequencing depth, low cDNA library complexity, and other technical factors impact the ability to fully integrate DNA sequencing with scRNA-seq.

## Quantifying Expressed Mutations in scRNA-seq

In principle, single-nucleotide variants (SNVs) and small insertions/deletions (INDELs) in expressed regions can be detected in scRNA-seq. In contrast to the detection of SNVs from exome sequencing (exome-seq), there are additional challenges inherent to quantifying SNVs in scRNA-seq (Piskol et al., 2013). Calling SNVs *de novo* from RNA sequencing (RNA-seq) is challenging, even from deeply sequenced bulk-RNA extractions. Variability in gene expression and allele-specific expression contribute significantly to the error rate (Castel et al., 2015). For scRNA-seq, these challenges are magnified by low coverage. Some scRNA-seq library prep protocols also impart additional coverage bias toward the 3′ end of the gene (Chapman et al., 2015), contributing to the dropout rate in SNV quantification in SNVs near the 5′ end. The most robust approaches to quantifying SNVs in single cells have integrated orthogonal data, to classify cells based on expressed mutations that were called first from DNA sequencing. For example, two recent studies combine scRNA-seq with exome-seq to map transcriptional signatures to inferred clones.

Kim et al. (2015) studied the effect of intra-tumor heterogeneity on anti-cancer drug-response using scRNA-seq and bulk exome-seq of patient-derived xenograft (PDX) tumor cells from a lung-adenocarcinoma patient. In a novel demonstration of the possibilities of single-cell data-integration, they correlated the presence of a KRAS mutation in individual cells to an expression signature characteristic of RAS/MAPK pathway activation. The study also revealed the technical limitations of quantifying SNVs in scRNA-seq. From more than 1,000 somatic SNVs identified via exome-seq, only 50 were expressed in more than three cells. Nonetheless, they did quantify a set of highly prevalent mutations affecting known oncogenes.

In another study, here of oligodendroglioma (Tirosh et al., 2016b), Tirosh and colleagues identified stem-like cells as the main source of tumor proliferation and the apex of a developmental hierarchy. To distinguish malignant from non-malignant cells, they developed a strategy to quantify the sensitivity of scRNA-seq in detecting somatic SNVs. The authors compare the variant-allele frequencies (VAFs) observed in exome-seq to the cellular frequencies of expressed mutations found in scRNA-seq. On average, somatic SNVs called from exome-seq could be validated in only 1.3% of the expected fraction of cells. Not surprisingly, the sensitivity of detection in scRNA-seq was positively correlated with gene expression levels. Ultimately, the authors found that they had much greater sensitivity in quantifying large-scale copy-number variants (CNVs), than they had with SNVs.

Large-scale CNVs are proving to be a genomic alteration that can be robustly quantified both in exome-seq (Alerting et al., 2012; Zack et al., 2013; Wang et al., 2015; Witkiewicz et al., 2015) and scRNA-seq (Patel et al., 2014; Müller et al., 2016; Tirosh et al., 2016a,b). While the expression level of an individual gene may be stochastically up- or down-regulated independent from its DNA copy-number, tumor/normal exome-seq read-count fold-changes correlate with single-cell expression-trendlines over megabase-scale regions (Peña-Llopis and Brugarolas, 2013; Hou et al., 2016; Müller et al., 2016). Moreover, by using a scRNA-seq data set from a relevant non-malignant tissue as a normal control, the error rate in quantifying the presence/absence of large-scale CNVs (called from exome-seq) in individual cells (assessed by scRNA-seq) can be rigorously controlled (Müller et al., 2016). It’s worth noting that large-scale CNVs are in principle detectible based on estimates of gene abundance alone, sequencing the entirety of each mRNA transcript is therefore not required. When large numbers of cells are sequenced simultaneously, cost-reduction strategies such as sequencing only the 3′ end of each mRNA are often employed. While most expressed SNVs and INDELs would be lost with 3′ sequencing, it is entirely compatible with large-scale CNV detection. All in all, for researchers who want to use scRNA-seq with heterogenous tumor samples, where neoplastic cells must be reliably separated from stromal and immune cells, augmenting scRNA-seq with exome-seq is a cost-effective strategy for achieving specificity while producing versatile data.

## Filtering and Classifying Stromal and Immune Cells from Whole-Tumor scRNA-seq

While bulk RNA-seq experiments can only estimate the fraction of stromal and immune cells (Yoshihara et al., 2013; Becht et al., 2016), scRNA-seq gives information about the identity of every cell sequenced (Wagner et al., 2016). Neoplastic cells can often be distinguished from stromal/immune cells via a clustering of gene expression profiles (Satija et al., 2015). However, some degree of stochastic mixing inevitably occurs when clustering cells by gene expression. Neoplastic cells can also express genes typically associated with immune cells, further adding to the ambiguity of classification via clustering alone (Patel et al., 2014).

The inference of large-scale CNVs from scRNA-seq data has become one of the most reliable techniques to distinguish neoplastic from stromal/immune cells (Tirosh et al., 2016a). For example, Tirosh et al. (2016b) used the presence of the 1p/19q co-deletion in oligodendrogliomas [a hallmark of that disease (Yip et al., 2012)] to identify neoplastic cells. Of the approximately 7% of cells that lacked detectable CNVs, all expressed markers of microglia or oligodendrocytes, confirming their approach. A related computational technique that can be used to add support to inferred, large-scale CNVs uses the VAFs of heterozygous germline mutations. Changes in copy number will skew the observed VAFs of heterozygous germline SNVs. Analysis of germline SNV VAFs is integrated into state-of-the-art algorithms to detect large-scale CNVs from exome-seq data (Favero et al., 2015), but its utility has not yet been explored in scRNA-seq data. As opposed to somatic SNVs, germline SNVs have been shown to have less allelic bias (Li et al., 2015). This suggests that germline-SNV VAF analysis can provide additional evidence to confirm large-scale CNVs.

**FIGURE 1:**
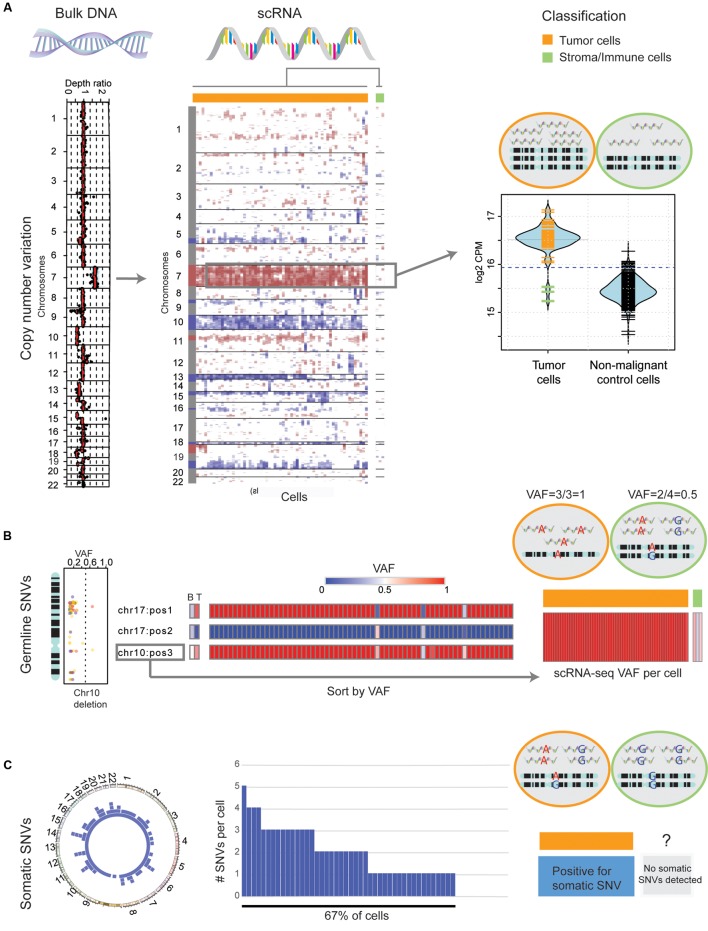
Classification of genomic mutations in single cells distinguishes neoplastic cells from immune infiltrate. **(A)** Left: The depth ratio of exome-seq reads from bulk tumor and blood control (*x*-axis) along autosomes (*y*-axis) identifies large-scale CNVs in a primary GBM. Middle: The detected genomic CNVs are reflected in single cells (columns) from the same case after normalizing the mean expression, within windows of 100 adjacent genes, by the mean expression in a normal brain control (red: fold-change > 1, blue: fold-change < 1). Hierarchical clustering (complete linkage, Euclidean distance) reveals three cells lacking large-scale CNVs. Right: A comparison of total sequencing depth on chromosome 7, measured by the sum-total counts per million (CPM), in individual cells between the tumor biopsy and a normal brain control. The 5% significance level of the control distribution is indicated by dotted lines. **(B)** Left: The VAF of heterozygous germline mutations (*x*-axis) deviates from 0.5 in regions of copy number alterations, here chromosome 10 is given as an example. Middle: Three heterozygous germline SNVs change in VAF (0.5 in blood sample) between blood (B) and tumor (T) exome-seq. In RNA-seq of individual cells, only the reference (blue) or the variant allele (red) are observed. Three cells are outliers, expressing both alleles. Right: The presence of both alleles in these three cells verifies their previous classification as non-neoplastic based on CNVs. **(C)** Left: Circos-plot of somatic SNVs detected by Mutect from exome-seq, for all autosomes. Middle: Histogram of somatic SNVs (*y*-axis) detected in single cells (*x*-axis). Right: 67% of cells can be classified as tumor cells due to the presence of at least one somatic SNV that has been validated in exome-seq.

Integrating an auxiliary exome-seq experiment provides a cost-effective way to rigorously separate neoplastic from stromal and immune cells, in scRNA-seq data. In this context, we propose separating cells based on four sources of evidence: (1) large-scale CNVs that are observed in both platforms; (2) the VAFs of germline SNVs, compared between platforms; (3) somatic SNVs found in both platforms; and (4) a clustering of scRNA-seq transcriptional profiles. As an example, we apply the above criterion to previously published scRNA-seq and matched exome-seq from a primary human glioblastoma (GBM) biopsy, SF10360 (Müller et al., 2016). Exome-seq revealed large-scale CNVs common in GBM, including a gain of chromosome 7 and a loss of chromosome 10 (Li et al., 2012). Both occurred with high VAF. Plotting gene expression, in sliding windows of 100 adjacent genes and normalized by a non-malignant brain control (Darmanis et al., 2015), indicates the presence of these two mutations in all but three cells (**Figure 1A**, middle). We previously described an approach to rigorously classify the presence of large-scale somatic CNVs in single cells, by comparison to a set of non-malignant control cells (Müller et al., 2016). These three cells show no evidence of CNVs, based on that method (**Figure 1A**, right). Next, consider heterozygous germline SNVs with differences in VAF between blood and tumor exome-seq. Cells harboring heterozygous germline SNVs in regions of copy-number loss should only express either the reference or the variant allele, thus providing further support for single-cell CNV calls. Three germline SNVs, two on chromosome 10 and one on chromosome 17 fulfill these criteria (**Figure 1B**, left). While there is only one allele found in putative neoplastic cells, the three cells which lack clonal, large-scale CNVs express both the reference and germline variants (**Figure 1B**, middle). Furthermore, of the somatic SNVs identified in exome-seq (**Figure 1C**, left), 67% of cells express at least one (**Figure 1C**, middle). Cells not classified as neoplastic (based on large-scale CNV and germline-SNV analysis) are devoid of somatic SNVs (**Figure 1C**, right), further confirming their status as non-neoplastic cells. Finally, hierarchical clustering in the space of GBM marker-genes as well as tumor-associated-macrophage markers reveals two clusters of cells (**Supplementary Figure S1**). The 3 putative non-malignant cells clustered separately and express high levels of macrophage/microglia markers. Taken together, we can classify these three cells as non-neoplastic, infiltrating immune cells based on our four criteria.

## Accessing Intra-Tumor Heterogeneity

**FIGURE 2:**
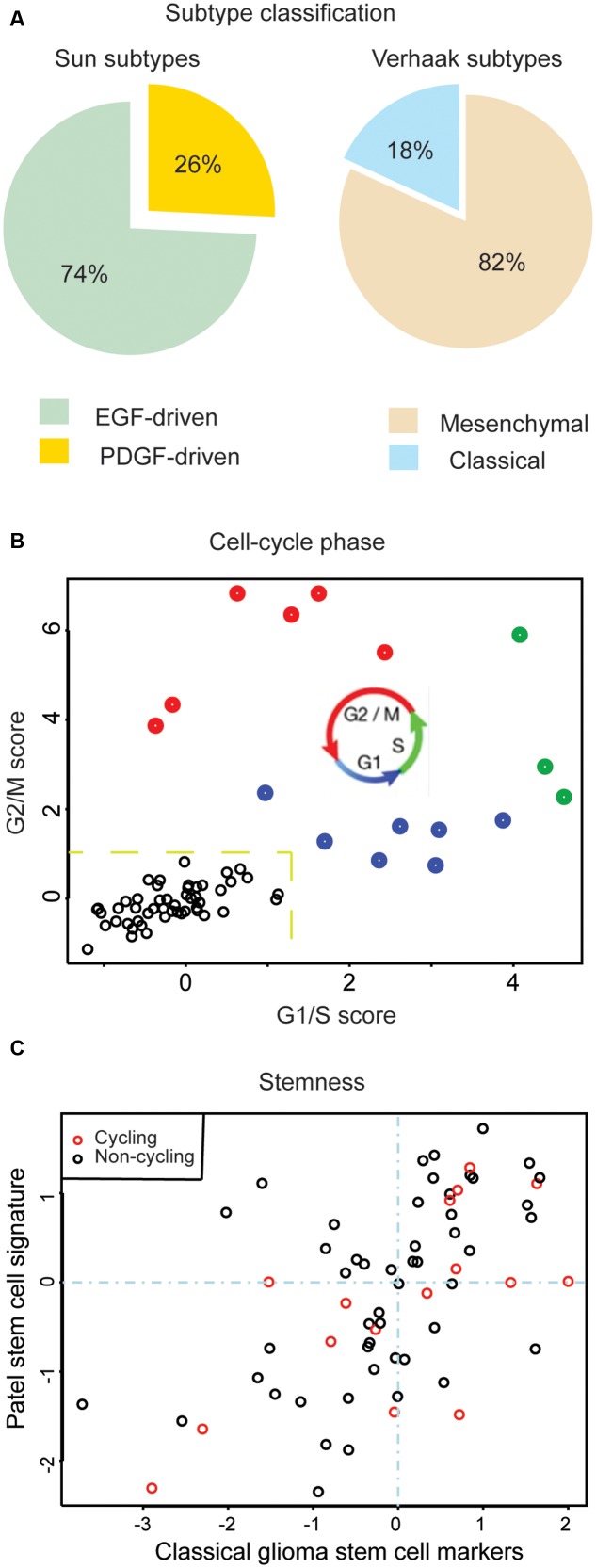
Assessments of intra-tumor heterogeneity made possible by scRNA-seq. **(A)** Percentage of single cells associated to a given GBM subtype. Adapted from Müller et al. (2016). **(B)** Estimation of cycling cells. An average of G1/S and G2/M scores > 1.2 classifies cells as cycling (labeled in red, green, or blue) or non-cycling (labeled in black). **(C)** Comparison of stem-like expression signatures for individual cells, based on marker genes canonical to GBM stem-cells: *CD44, CD133 (PROM1), NES, KLF4, MYC, NANOG, STAT3, SOX2, MET* (*x*-axis) and marker genes published by Patel et al. (2014) (*y*-axis).

Large-scale molecular profiling has identified prognostic cancer-subtypes based on transcriptional signatures (Brennan et al., 2013; Cancer Genome Atlas Research Network, 2013, 2014a,b; Bass et al., 2014; Cancer Genome Atlas Research Network, et al., 2016; Wang Q. et al., 2016). However, recent scRNA-seq studies have revealed that most tumors are a heterogeneous composition of cells conforming to multiple subtypes (**Figure 2A**) (Patel et al., 2014; Müller et al., 2016). Since a variety of genomic alterations are detectible in scRNA-seq data, scRNA-seq can be used to analyze intra-tumor heterogeneity at both the transcriptional and mutational levels simultaneously. This is useful for studying how intra-tumor heterogeneity arises in the first place. Several groups have begun to use scRNA-seq data to address the fundamental question of how tumors propagate through cellular hierarchies (Müller et al., 2016; Tirosh et al., 2016b; Woodworth et al., 2017).

In the cancer stem-cell model, a small population of stem-like cells gives rise to differentiated, phenotypically diverse progeny with limited proliferative potential (Ghaffari, 2011). Assuming that the majority of these cancer stem-cells persist in a slow-cycling or quiescent state, as observed in some cancers (Dembinski and Krauss, 2010; Chen et al., 2012), the genetic diversity of the tumor is largely explained by the genetic diversity within the stem-cell population. In the model of clonal evolution, those acquired mutations which provide a selective advantage will expand (Greaves and Maley, 2012). These two models are not strictly contradictory. The progeny of cancer stem-cells may retain proliferative potential and thereby contribute additional mutations. If cancers follow the stem-cell model, clonal evolution, or a mixture of both, or if this even depends on the cancer type currently remains an open question (Shackleton et al., 2009). ScRNA-seq is uniquely suited to address this challenge. Two recent studies have performed this type of integrated analysis, both in glioma.

Working with high-grade glioblastomas, Müller et al. (2016) first identified large-scale CNVs from exome-seq data and then classified individual cells according to the presence or absence of these alterations via scRNA-seq. Using standard phylogenetic approaches, they then organized cells into mutational hierarchies. They found that these hierarchies correlated with transcriptional hierarchies of cell-types found in the developing brain. Tirosh et al. (2016b) took a complementary perspective and first organized their low-grade glioma scRNA-seq data based on hierarchies of transcriptional phenotypes, corresponding to stem cells and their differentiated progeny. They then cross-referenced validated, expressed mutations. In contrast to Müller et al. (2016) they found that their transcriptional and mutational hierarchies were largely uncorrelated. While in Müller et al. (2016) found that differentiated cell types more frequently harbored sub-clonal mutations then stem-like cells, Tirosh et al. (2016b) found that sub-clonal mutations occurred with equal frequency in both stem-like and differentiated populations. The interpretation of Tirosh et al. (2016b) was that in their low-grade gliomas proliferation was restricted to stem-like cells. By contrast, the data of Müller et al. (2016) support an expansion in high-grade glioblastoma of proliferative cell-types that do not have a stem-like transcriptional signature, but rather the mRNA profile of an oligodendrocyte progenitor or migrating neuroblast. An expansion of transit-amplifying, proliferative cell-types in high-grade glioblastoma, relative to low-grade glioma, is also supported by a cell-cycle analysis of the scRNA-seq expression signatures. For example, in the glioblastoma case SF10360 described in Müller et al. (2016) cycling cells can be immediately identified and classified by cell-cycle stage (**Figure 2B**). Cycling glioblastoma cells are frequently depleted of both the glioma-stemness genes identified by Patel et al. (2014), as well as classical glioma stem cell markers (**Figure 2C**) (Bradshaw et al., 2016). This type of analysis, where cells are separated based on genomic alterations, transcriptional phenotypes (e.g., stem-like expression pattern), or cell state (e.g., cycling cells), demonstrates the versatility of scRNA-seq data.

## Predicting of Interactions Between the Tumor and the Microenvironment

**FIGURE 3:**
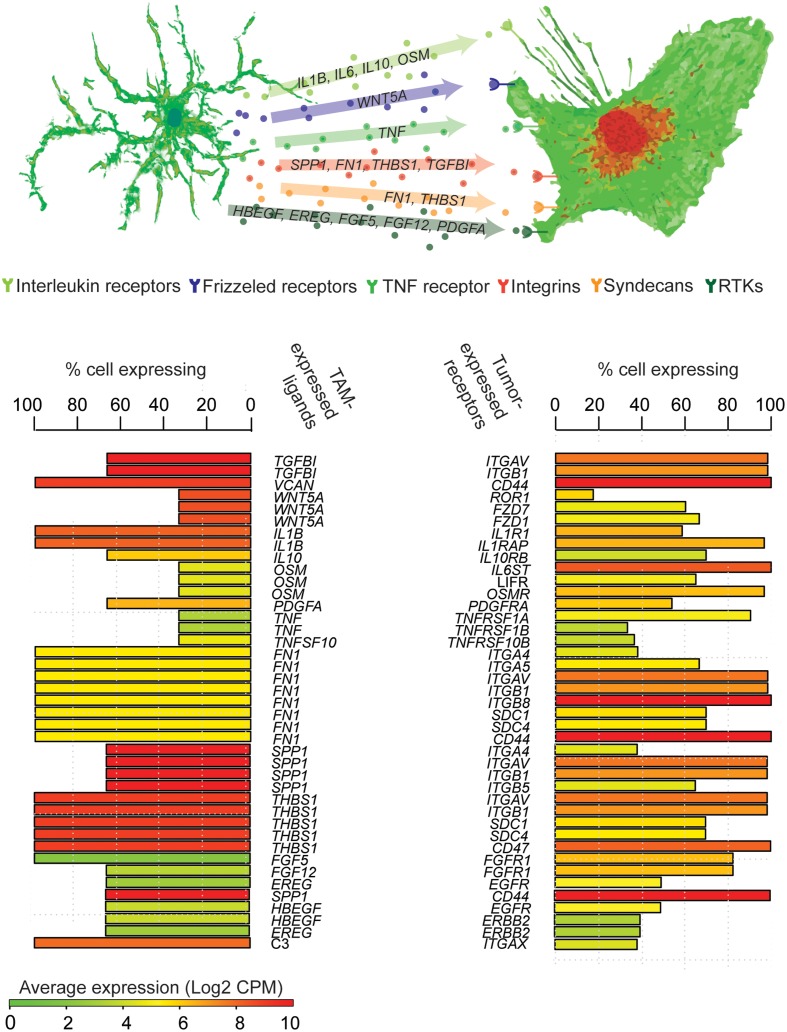
Inference of TAM-tumor crosstalk from scRNA-seq. Genes encoding ligands robustly expressed by at least 20% of TAMs with an average expression >2 CPM are paired with genes encoding their cognate receptors that are expressed in tumor cells. Each row represents a potential tumor-TAM interaction, bars represent the percentage of cells expressing each mRNA, colors indicate mean expression across cells.

Tumor-infiltrating stromal and immune cells contribute significantly to tumor heterogeneity (Augsten, 2014). While computational models for predicting tumor-stroma crosstalk from bulk-extraction sequencing experiments are under development (Hackl et al., 2016), scRNA-seq also provides a powerful tool to infer paracrine-signaling networks. For example, in glioma, tumor associated macrophages/microglia (TAMs) are the most abundant immune infiltrate and can reach up to 30% of the total tumor mass (Cretu et al., 2005). By simply cross-referencing gene expression levels in single TAMs and neoplastic cells sequenced from SF10360 (Müller et al., 2016), with the receptor-ligand pairs from CCCExplorer (Choi et al., 2015), one can infer a myriad of potential crosstalk (**Figure 3**). Here we see that TAMs express a variety of growth factors and growth-promoting cytokines, while neoplastic cells from the same sample express their cognate receptors. ScRNA-seq thus provides a powerful hypothesis-generating mechanism for paracrine-signaling studies.

## Conclusion

Recent advances in scRNA-seq have led to novel insights in cancer development, progression, metastasis, and drug-resistance, that were previously “veiled” by the mixing of cells intrinsic to standard bulk-sequencing experiments. Still, a variety of challenges go hand in hand with this rapid progress. For example, reliably distinguishing between neoplastic and infiltrating stromal/immune cells requires more than an analysis of transcriptional profiles alone. Analysis of expressed SNVs, CNVs, and other mutations from scRNA-seq can be used to filter stromal from neoplastic cells, and to map gene-expression signatures to putative tumor sub-clones. While most cancer scRNA-seq studies to date have focused on tumor cells, applications of scRNA-seq to paracrine-signaling studies of the tumor microenvironment are an exciting frontier. Therefore, scRNA-seq is a powerful tool for understanding the molecular processes that govern one of the most difficult diseases of our time: Cancer.

## Author Contributions

SM and AD wrote the manuscript. SM collected literature and generated figures with input from AD.

## Conflict of Interest Statement

The authors declare that the research was conducted in the absence of any commercial or financial relationships that could be construed as a potential conflict of interest.

## Supplementary Material

**FIGURE S1 |** Expression profiles support mutation-based cell classification. Expression of immune and tumor marker-genes (rows) in individual cells (columns). Clusters obtained via unsupervised hierarchical clustering are indicated by black bars.
