# Supplementary figures and images for: Single-Cell mRNA Sequencing in Cancer Research: Integrating the Genomic Fingerprint
Source: Front Genet. 2017 May 31;8:73. doi: 10.3389/fgene.2017.00073 (PMC5450061; doi:10.3389/fgene.2017.00073)

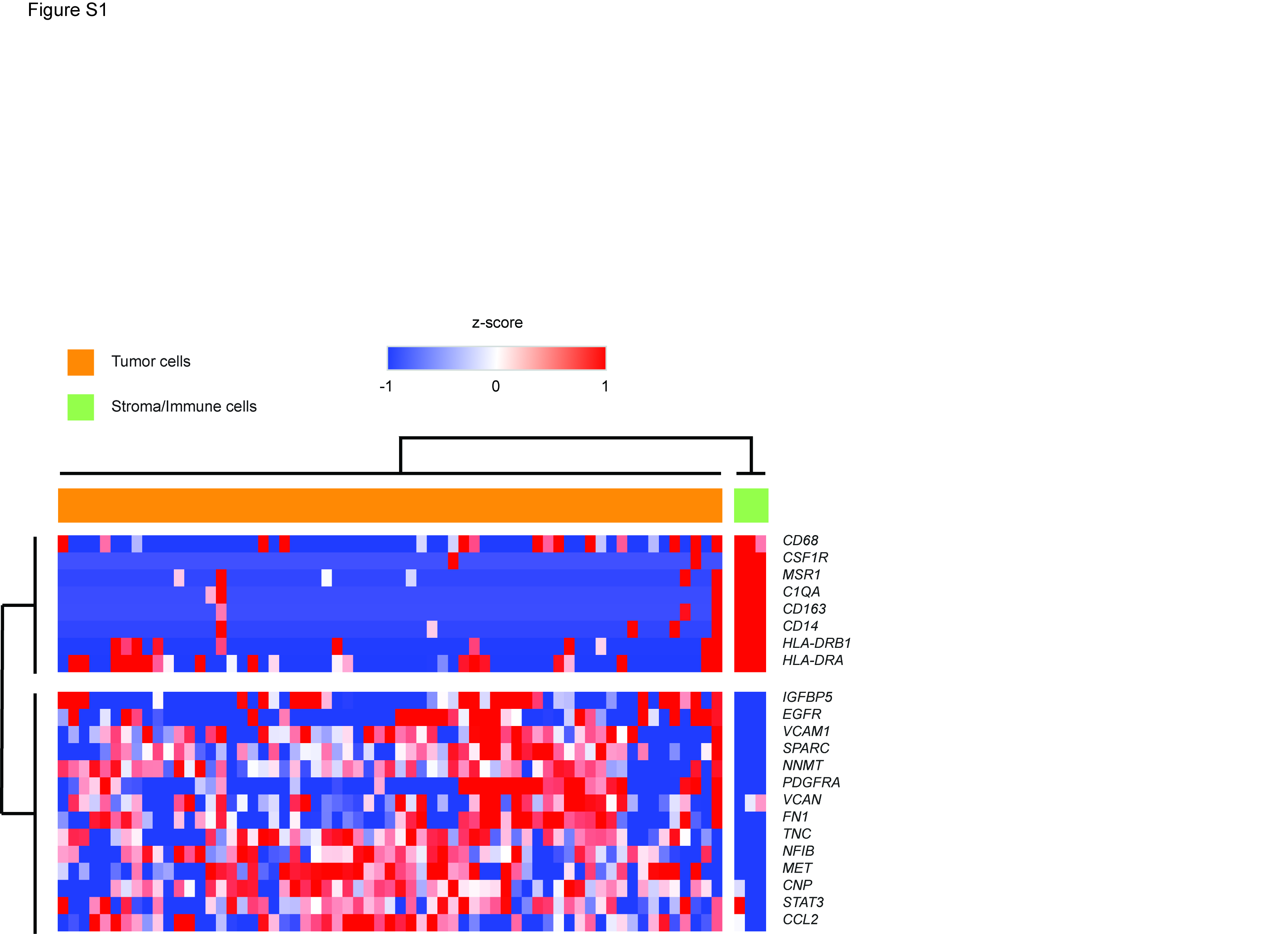

Supplement: Supplementary file 1 [file Image_1.tif]
